# Retraction Note: Enhanced climate change resilience on wheat anther morphology using optimized deep learning techniques

**DOI:** 10.1038/s41598-026-40318-8

**Published:** 2026-02-18

**Authors:** Arifa Zahir, Zulfiqar Ali, Ahmad Sami Al-Shamayleh, Syed Raza Ab bas, Basharat Mahmood, Abdullah Hussein Al-Ghushami, Rubina Adnan, Adnan Akhunzada

**Affiliations:** 1 Department of Bioscience, COMSATS University, Islamabad, 45550 Pakistan; 2 Department of Computer Science, COMSATS University, Islamabad, 45550 Pakistan; 3 Department of Data Science and Artificial Intelligence, Faculty of Information Technology, Al-Ahliyya Amman University, Amman, Jordan; 4 Department of Information Technology, Community College of Qatar, Doha, Qatar; 5 College of Computing & IT, University of Doha for Science and Technology, Doha, Qatar

Retraction of: *Scientific Reports* 10.1038/s41598-024-74875-7, published online 19 October 2024

The Authors have retracted this Article as they could not reach agreement on data ownership. The data used in this study originated from the thesis work of the first author and was supervised by a researcher who is not listed as an author and who did not grant permission for the use of the data.

All Authors agree with this retraction.

